# Association between periodontal disease and prostate cancer: a systematic review and meta-analysis

**DOI:** 10.4317/medoral.24308

**Published:** 2020-11-28

**Authors:** Yali Wei, Yongjin Zhong, Yan Wang, Ruijie Huang

**Affiliations:** 1 State Key Laboratory of Oral Diseases, National Clinical Research Center for Oral Diseases, Dept. of Pediatric Dentistry, West China Hospital of Stomatology, Sichuan University. P. R. China

## Abstract

**Background:**

Periodontal disease is a chronic infectious disease caused by bacterial infection which may lead to various systematic diseases. Recently, increasing studies have explored the correlation of periodontal disease with the risk of prostate cancer. However, the findings were inconsistent. Hence, this study aims to investigate the association between periodontal disease and the risk of prostate cancer by a meta-analysis.

**Material and Methods:**

PubMed, EMBASE, and Cochrane were searched for publications up to July 17, 2020. Cohort and case-control studies evaluating the risk of prostate cancer in patients with periodontal disease were included. A fixed or random-effect model was used to calculate the summary relative risk (RR) along with 95% confidence interval (CI). All analyses were conducted using Stata 12.0 software.

**Results:**

Seven studies were included in the final analysis. The pooled estimates showed that periodontal disease was significantly associated with the risk of prostate cancer (RR = 1.17; 95% CI = 1.07-1.27; *P* = 0.001). Findings of sensitivity analyses proved that the overall results were robust.

**Conclusions:**

Periodontal disease may be considered as a potential risk factor for prostate cancer. Although it’s a possibility, males should be more aware of their oral health and implement effective measures to prevent and treat periodontal disease.

** Key words:**Periodontal disease, periodontitis, prostate cancer, meta-analysis

## Introduction

Prostate cancer (PC) composes the largest part of the diseases that affect men’s health worldwide, and its incidence increases with age (1). It is currently the most common cancer diagnosed in males living in developed nations and is becoming more commonly encountered in the rest of the world (2). According to statistics, PC accounts for 20% of new male cancers in the United States in 2019 (3).

Currently, numerous epidemiologic studies have linked the risk of PC to various factors like age, ethnicity, family history, insulin-like growth factors, lifestyle, diet, environmental and occupational exposures (4,5). Besides, Kim *et al*. (6) also reported that chronic inflammation was the cause of PC, playing a vital role in tumor initiation, promotion, malignant transformation, invasion and metastasis.

Periodontal disease (PD) is a chronic infectious disease caused by bacterial infection which invades gingiva and periodontal supporting tissues (7). It affects 47 % of adults aged 30 and older in the United States, and leads to gradual loss of periodontal tissues including periodontal bone, and in aggressive and severe cases to tooth loss (8,9). The prevalence and severity of PD, especially periodontitis, increase with age (10). Many studies have thus paid attention to the relationship between PD and other diseases such as Alzheimer's disease and neuroinflammation (11,12). In recent years, many studies have confirmed that PD may increase the incidence of certain cancers such as lung cancer, colorectal cancer, breast cancer, head and neck cancer, which may be related to the increased inflammation of PD (13,14).

At present, although some studies have found a close relationship between PD and PC (15-21), the results of these studies are inconsistent. Therefore, the purpose of this study lies on conducting a meta-analysis to assess and quantify the relationship between PD and the risk of PC.

## Material and Methods

This meta-analysis was based on the Preferred Reporting Items for Systematic Review and Meta-analysis (PRISMA) guidelines (22).

- Eligibility criteria

Studies meeting the following criteria were included: 1) cohort study or case-control study; 2) the exposure of interest was PD, and the comparison was an absence of PD; 3) the outcome of interest was patients with PC; 4) the hazard ratio (HRs), relative risk (RRs) or odds ratio (ORs) estimates with their 95% confidence intervals (CIs) were reported; 5) the study with higher quality or better comprehensiveness was included if two or more articles used the same dataset; 6) studies were published in English.

- Search strategy

A comprehensive literature search was conducted with PubMed, EMBASE, and Cochrane to identify studies up to July 17, 2020, using the following search terms: (“prostate neoplasm” OR “prostate cancer” OR “prostate tumor” OR “prostatic cancer” OR “prostatic neoplasm” OR “prostatic tumor”) AND (“periodontal disease” OR parodontopathy OR parodontosis OR paradontosis OR paradontopathy OR periodontal OR periodontium OR periodontitis OR “periodontal attachment loss” OR “periodontal pocket” OR “alveolar bone loss” OR “gum disease” OR “pyorrhea alveolaris” OR periodontitides OR pericementitis OR pericementitides). No data and language restrictions were applied in searching. Additional studies were retrieved by searching reference lists of relevant articles.

- Study selection and data extraction

Duplicate records were removed, then study titles and abstracts were screened to make sure they were relevant. Subsequently, studies that satisfied the eligibility criteria were included through full-text assessment.

The following data were obtained from each included study: first author’s surname, year of publication, country of origin, study design, sample size, assessment methods of exposure and outcome, age, follow-up period, the estimate (HR, RR or OR) with 95% confidence interval (CI) and adjustment confounding factors. These data were independently extracted by two authors (YW and YZ). Any disagreements between these authors were resolved through discussion or by consulting a third author (RH).

- Quality assessment

The methodological quality of the included studies was assessed by Newcastle-Ottawa Scale (NOS) (23). The scale consists of three categories with a total score of nine points: selection, comparability and exposure/outcome. A score below 4 was defined as low quality, 4-6 points as mean moderate quality, while 7-9 points represented good quality. Two authors (YW and YZ) independently evaluated the quality of the included studies. Discrepancies were discussed with the third author (RH) until a consensus was reached in the end.

- Statistical analysis

RR with 95% CI was used as the pooled estimate to assess the association of PD and the risk of PC. When the outcome of interest was rare, it was considered that the OR approximated the RR (24). HR was also treated as RR when pooled in this meta-analysis. The heterogeneity across studies was evaluated by Q test (Statistical significance was considered when *P* < 0.1) and the I2 statistic (I2 ≥ 50% indicated significant heterogeneity). In the presence of significant heterogeneity, the random effect model was adopted; otherwise, the fixed model was used. Subgroup analyses stratified by study design, country of origin, follow-up period, sample size and quality of studies were conducted. Sensitivity analysis was performed to examine the robustness of the results by using a leave-one-out approach. Begg’s test and Egger’s test were used to assess the Publication bias. A P-value of less than 0.05 was regarded statistically significant. All analyses were conducted with Stata 12.0 software (Stata, version 12; StataCorp, College Station, TX, United States).

## Results

- Study selection and characteristic

**Figure 1:**
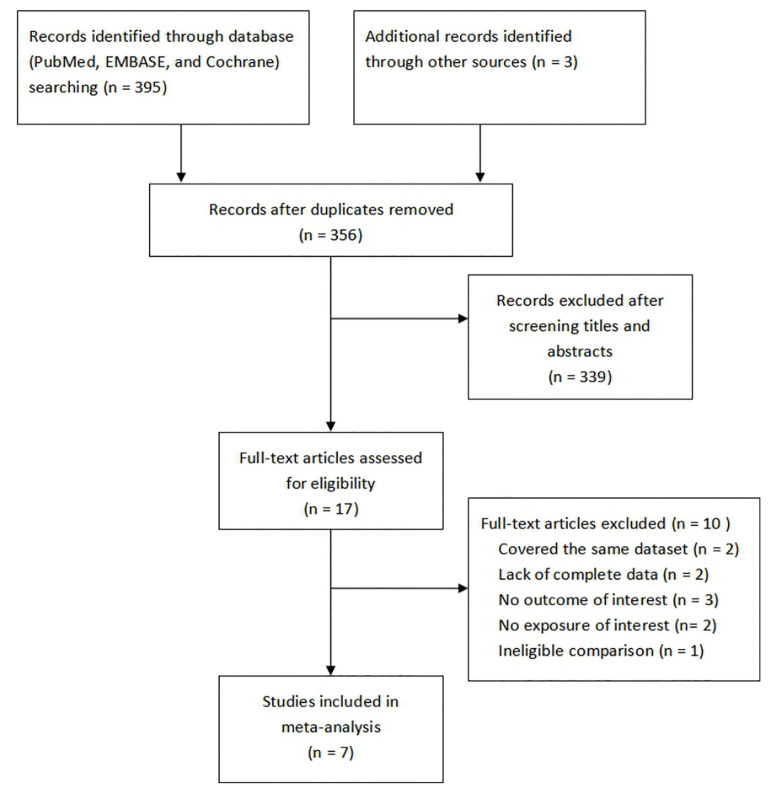
Flow chart of search process and reasons for exclusion.

A total of 398 studies were initially identified. After excluding 42 duplicates and 339 articles through screening titles and abstracts, 17 full-text articles were retrieved for further assessment. Seven studies were included in the end to the qualitative synthesis after removing the studies that had no outcome of interest (n = 3), exposure of interest (n = 2), complete data (n = 2), eligible comparison (n = 1) nor overlaps in datasets (n = 2) (Fig. 1). Of the included studies, four were prospective cohort studies (15,18,20,21) and three were retrospective cohort studies (16,17,19). Three studies were conducted in the United States (18,20,21), two studies were carried out in Europe (15,17), and two studies were performed in Asia (16,19). However, only one of these studies explored the correlation between severity degrees of PD and the risk of PC (21). All studies provided adjusted estimates and corresponding 95% confidence intervals. In addition to the characteristics of included studies, Table 1 consists of the results of their quality assessment according to the NOS. All studies were of high quality except the study by Chung *et al*. (16) which was considered to be of moderate quality. This study was not eliminated from the meta-analysis nevertheless since it had no influence on the pooled estimates (the results are presented in the sensitivity analysis section).

- Overall result and subgroup analysis

**Figure 2:**
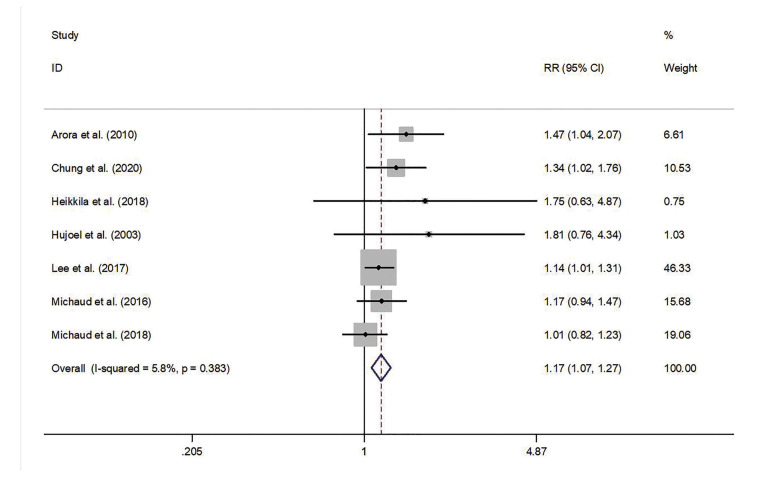
Forest plot of PD and the risk of PC.

Pooled estimates indicated that PD had a significant statistical relationship with the risk of PC (RR = 1.17; 95% CI = 1.07-1.27; *P* = 0.001). No significant heterogeneity across studies was observed (*P* = 0.383; I2 = 5.8%; Fig. 2).

Subgroup analysis was stratified by the study design, follow-up period, country of origin, sample size and quality of studies. The results of the subgroup analysis are demonstrated in Table 2.

**Table 1:**
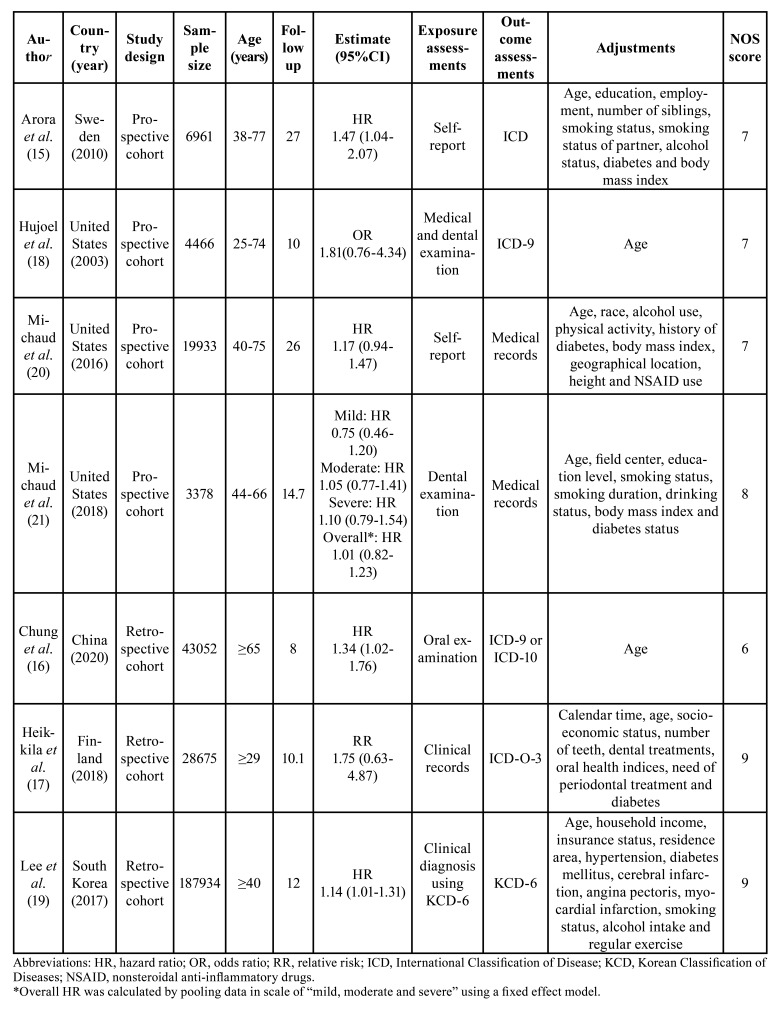
Characteristics of the included studies on the association between PD and PC.

**Table 2:**
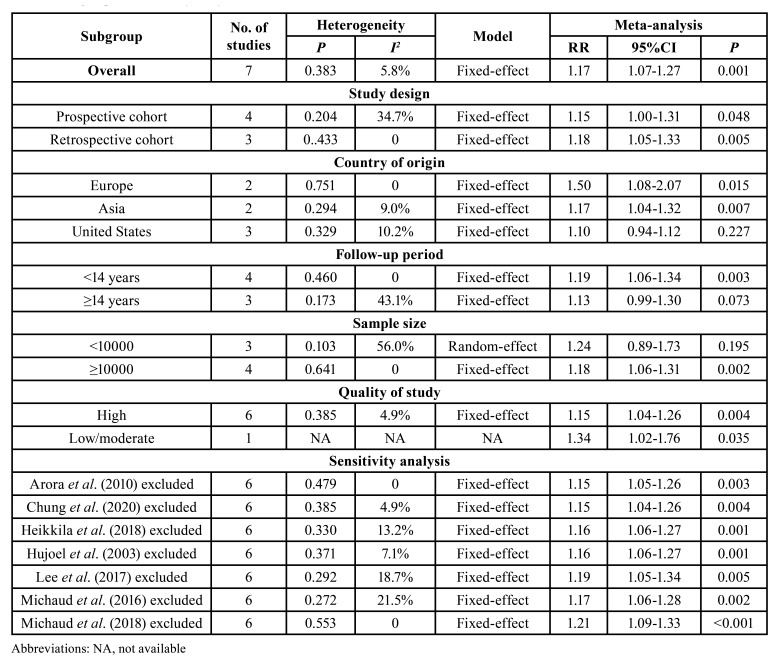
Subgroup and sensitivity analyses of the association between PD and the risk of PC.

The pooled estimates of the subgroup analysis on the basis of study design showed a significant association between PD and the risk of PC in three retrospective cohort studies (RR = 1.18; 95% CI = 1.05-1.33) and four prospective cohort studies (RR = 1.15; 95% CI = 1.00-1.31). For analyses based on the country of origin, the association of PD and the risk of PC was statistically significant among Asian populations (RR = 1.17; 95% CI = 1.04-1.32) or European populations (RR = 1.50; 95% CI = 1.08-2.07) but not among American populations (RR = 1.10; 95% CI = 0.94-1.12). In terms of the duration of follow-up, the pooled results of four studies which followed target populations of < 14 years showed a significant relation between PD and the risk of PC (RR = 1.19; 95% CI = 1.06-1.34); no evidence of increased risk of PC among patients with PD was found in the studies with a follow-up period of ≥ 14 years (RR = 1.13; 95% CI = 0.99-1.30). Analyses according to sample size revealed that PD was associated with the risk of PC in the studies with less than 10,000 participants (RR = 1.18; 95% CI = 1.06-1.31) but not in the studies with more than 10,000 participants (RR = 1.24; 95% CI = 0.89-1.73). Moreover, after the exclusion of the study which was not of high quality, the results nevertheless indicated a significant association between PD and the risk of PC (RR = 1.15; 95% CI = 1.04-1.26).

- Sensitivity analysis

We deleted a study from the overall meta-analysis each time to explore the influence of the removed data on the pooled RR. The results showed omission of any individual studies brought no significant changes on pooled RR with 95% CI, indicating the meta-analysis results were robust (Table 2).

- Publication bias analysis

The results of Begg’s test (*P* = 0.230) and Egger’s test (*P* = 0.078) indicated there was no statistically significant publication bias across those studies.

## Discussion

To our knowledge, meta-analysis has not been used previously to explore the correlation of PD with the risk of PC. The pooled results suggested that PD was significantly associated with the risk of PC. Patients with PD have 1.17-fold increased risk of PC. Sensitivity analyses confirmed the robustness and reliability of our findings.

At present, certain underlying mechanisms suggest that there may be a causal relationship between PD and PC. Researchers generally believe that PD causes cancer mainly by affecting systemic immune response which leads to the increase of serum inflammatory markers, and moreover, the level of C-reactive protein particularly increases with the progress of PD (25). Also, Joshi *et al*. has found that the level of prostate-specific antigen (PSA), used to diagnose PC, was significantly higher in patients with PD (26). Some studies have proved the relationship between certain oral pathogens and the onset or progression of systemic cancer (12,27). Meurman *et al*. found that oral pathological bacteria are able to up-regulate or down-regulate pro-inflammatory cytokines and chemokines which affect oral and systemic immune system of the body, causing carcinogenic reactions (27). *Porphyromonas *gingivalis** and *Actinobacillus actinomycetemcomitans* were proven to be associated with a high risk of pancreatic cancer (28). However, the cause-effect relationship between PD and PC needs more studies to be confirmed since Hwang *et al*. (29) have reported that PD with the treatment cohort has not shown decrease in the risk of PC.

The meta-analysis performed by the authors of this paper consists of the following attributes: First, low heterogeneity across studies was observed, and there was no publication bias in the analyses. Second, the majority of studies included in the meta-analysis were considered to be of high quality according to the NOS. Although one of the studies was of moderate quality, no evidence of unsTable results was observed in the sensitivity analysis when excluded. Third, the selection and recall bias were minimized since all studies in our analyses were cohort studies. Fourth, as most human cancers show a long latent period, it is necessary to have a long duration of follow-up designed for studies. In this meta-analysis, most studies had more than 10 follow-up duration, and three of them were with a follow-up period of more than 14 years.

Despite these strengths, there were several limitations of this meta-analysis. First, according to the results of subgroup analysis, the estimates from the subset of studies on target populations from the United States with longer follow-up or smaller sample size indicated that PD had no significant association with the risk of PC, which meant several factors may affect the correlation between PD and the risk of PC. The interpretation was yet unconvincing due to the limited number of studies in each subgroup. Second, although the majority of confounding factors were adjusted in the included studies, few studies adjusted for dental health status, stress and occupation, which may influence the precision of analyses. Third, it is clear that familial and genetic risk factors lead to the increased risk of PC (30), however, analyses were not conducted due to the lack of relevant data. Fourth, the assessment methods of PD were different among the included studies. Two studies used self-reports from participants to evaluate PD (15,20), four studies assessed PD status using the result of a dental examination or clinical diagnosis (16,18,19,21), and one study used the records from dentists (17). This may become a source of heterogeneity, influencing the overall estimates. Lastly, since only one study reported the stages of PD (21), it was difficult to further investigate the different correlations between the patients with various degree of PD (mild, moderate or severe) and the risk of PC.

## Conclusions

In summary, the present meta-analysis revealed that PD is significantly associated with the risk of PC, and that PD may increase the risk of PC. Based on the results, the general public should be more aware of their periodontal health and maintain a good oral health which may be beneficial in reducing possible risk of PC. However, well-designed studies with a more extended follow-up period, better assessment methods of PD, populations from diverse geographic regions and solid multivariable adjustments are warranted to confirm our findings.
